# Immunophenotype and molecular characterisation of adenocarcinoma of the small intestine

**DOI:** 10.1038/sj.bjc.6605449

**Published:** 2009-11-24

**Authors:** M J Overman, J Pozadzides, S Kopetz, S Wen, J L Abbruzzese, R A Wolff, H Wang

**Affiliations:** 1 Department of Gastrointestinal Medical Oncology, The University of Texas M. D. Anderson Cancer Center, 1515 Holcombe Boulevard, Houston, TX 77030, USA; 2 Department of Internal Medicine, The University of Texas Health Science Center at Houston, 6431 Fannin, Suite MSB 1.150, Houston, TX 77030, USA; 3 Department of Biostatistics, The University of Texas M. D. Anderson Cancer Center, 1515 Holcombe Boulevard, Houston, TX 77030, USA; 4 Department of Pathology, The University of Texas M. D. Anderson Cancer Center, 1515 Holcombe Boulevard, Houston, TX 77030, USA

**Keywords:** small bowel adenocarcinoma, immunohistochemistry, mismatch repair, epidermal growth factor receptor, vascular endothelial growth factor

## Abstract

**Background:**

Despite having a dramatically larger surface area than the large intestine, the small intestine is an infrequent site for the development of adenocarcinoma. To better understand the molecular abnormalities in small bowel adenocarcinoma (SBA), we characterised a number of candidate oncogenic pathways and the immunophenotype of this rare cancer.

**Methods:**

Tissue microarrays were constructed from tumour samples from 54 patients with all stages of the disease. Immunohistochemistry and microsatellite instability (MSI) testing were conducted.

**Results:**

The profile of cytokeratin 20 and 7 coexpression was variable, but expression of caudal type homeobox transcription factor 2 (CDX2) was present in 70% of cases. In this young population (median age 54 years), loss of mismatch repair (MMR) proteins occurred in 35% of patients, with confirmed MSI in 100% of tested cases. Expression of vascular endothelial growth factor-A (VEGF-A) and epidermal growth factor receptor (EGFR) was common, occurring in 96 and 71% of patients, respectively. Only one case showed HER2 expression and none showed loss of phosphatase and tensin homologue mutated on chromosome 10 (PTEN).

**Conclusions:**

These results suggest that alterations in DNA MMR pathways are common in SBAs, similar to what is observed in large bowel adenocarcinomas. Furthermore, the high percentage of tumours expressing both EGFR and VEGF suggests that patients with this rare cancer may benefit from therapeutic strategies targeting EGFR and VEGF receptor (VEGFR).

Adenocarcinoma of the small bowel is a rare, aggressive malignancy with poor overall outcome. The majority of patients with small bowel adenocarcinoma (SBA) present with advanced disease, with 5-year disease-specific survival rates of 35% for patients with lymph node involvement and 4% for patients with metastatic disease (Howe *et al*, 1999). Although the small intestine makes up approximately 75% of the length and 90% of the mucosal surface of the gastrointestinal tract, SBA occurs 50 times less frequently than colorectal adenocarcinoma (O'Riordan *et al*, 1996; DeSesso and Jacobson, 2001). Despite this intriguing biological difference in the incidence of SBA and colorectal adenocarcinoma, few investigations into the mechanisms of small bowel carcinogenesis have been conducted. A number of theories have been postulated to explain the relative protection of the small intestine from the development of carcinoma; however, none have been proven. Proposed protective factors have generally centred around two concepts. First, the rapid turnover of small intestinal cells results in epithelial cell shedding before the accumulation of the genetic damage critical to carcinogenesis. Second, exposure of the small intestine to the carcinogenic components of our diet is limited because of the small intestine's rapid transit time, lack of bacterial degradation activity, and relatively dilute alkaline environment.

As in colorectal cancer, a subset of SBAs are characterised by a defect in DNA mismatch repair (MMR), which results in DNA microsatellite instability (MSI) (Planck *et al*, 2003). Microsatellite instability is characterised by the accumulation of changes in the length of simple repeated nucleotide sequences known as microsatellites, caused by mutations in MMR genes such as MutS homologue 2 (hMSH2), MutL homologue 1 (hMLH1), post meiotic segregation increased 1 (hPMS1), hPMS2, and hMSH6. Although MSI is a hallmark of hereditary nonpolyposis colorectal cancer syndrome, in which mutations of one or more MMR genes are found in >90% of the cases, it has also been reported in approximately 10% of sporadic colorectal adenocarcinomas (Liu *et al*, 1995). In colorectal cancer, defects in DNA MMR are characterised by a younger age of onset, poorly differentiated mucinous tumours, and improved outcomes (Gryfe *et al*, 2000; Jenkins *et al*, 2007). However, for patients with SBA, no comprehensive study has been conducted to compare the clinical and pathological differences between patients with intact and deficient DNA MMR.

Other oncogenic signalling pathways that are active in colorectal cancer include the epidermal growth factor receptor (EGFR), vascular endothelial growth factor receptor (VEGFR), and phosphatidylinositol 3-kinase (PI3K)/AKT pathways. Epidermal growth factor receptor is a member of the ErbB family of transmembrane receptor tyrosine kinases that have a crucial role in tumour cell proliferation, survival, adhesion, migration, and differentiation (Yarden and Sliwkowski, 2001). Although the expression of EGFR has not been evaluated in SBA, one study has evaluated the expression of the ErbB family member, HER2. In this study, 9 of 16 patients showed HER2 expression and such expression was associated with a worse overall survival (OS; Zhu *et al*, 1996). Vascular endothelial growth factor signalling has a key role in tumour-associated neo-angiogenesis and the PI3K/AKT pathway is a critical pro-survival and pro-proliferation pathway that is frequently activated in a variety of cancers (Stambolic *et al*, 1998). The phosphatase and tensin homologue mutated on chromosome 10 (PTEN) protein acts as a potent tumour suppressor of this pathway and loss of PTEN expression is observed in approximately 5% of colorectal cancer (Zhou *et al*, 2002; Goel *et al*, 2004). Currently, no studies have evaluated the expression of EGFR, VEGF-A, or PTEN in SBA.

In addition, the published data on the immunophenotype of SBA are limited and conflicting (Lee *et al*, 2003; Chen and Wang, 2004). To date, no study has evaluated the expression of caudal type homeobox transcription factor 2 (CDX2) in a large number of patients with SBA. It is critical for the development and differentiation of the intestines, and highly expressed in cancers of the large intestine (Moskaluk *et al*, 2003; Werling *et al*, 2003).

To characterise the immunophenotype and the expression of a number of candidate oncogenic proteins, we performed immunohistochemical analysis of tumour samples from a large data set of SBA patients treated at The University of Texas M.D. Anderson Cancer Center. As a number of the proteins analysed represent therapeutic targets for drugs currently in clinical use, the results of this study have direct clinical relevance for the potential treatment of this rare cancer.

## Materials and methods

### Study population and tumour samples

We searched the M.D. Anderson Cancer Center pathology database from 1981 to 2007 for cases of SBA with accompanying pathologic materials. A total of 83 cases were identified and 54 cases were included. In all, 23 cases with inadequate pathologic materials and six cases of ampullary adenocarcinoma were excluded. The haematoxylin and eosin (H&E)-stained slides from all 54 cases were reviewed by a gastrointestinal pathologist (HW) to confirm the diagnosis and tumour grades. Normal small bowel mucosa samples were present in all but 12 of the cases. We retrospectively collected clinical and follow-up data, including demographics, cancer treatment history, stage, and survival for each patient from patient records. Tumour samples were from the primary site of resection in 43 cases and from a metastatic site in 11 cases. A total of 10 patients received anticancer therapy before collection of the pathologic material, with therapy consisting of chemoradiation in three cases and systemic chemotherapy in seven cases.

This study was carried out in accordance with the guidelines of the institutional review board.

### Tissue microarray

To construct the tissue microarray, the formalin-fixed, paraffin-embedded archival tissue blocks and their matching H&E-stained slides were retrieved, reviewed, and screened for representative tumour regions and normal small bowel mucosa by a gastrointestinal pathologist (HW). For each patient, three cores of tumour and when available, two cores of paired normal small bowel mucosa, were sampled from representative areas using a 1.0-mm punch. The tissue microarray was constructed with a tissue microarrayer (Beecher Instruments, Sun Prairie, WI, USA) as described previously (Wang *et al*, 2002). The constructed TMA blocks were sealed with paraffin, and 5-*μ*m-thick slides were cut from the TMA blocks for immunohistochemical staining.

### Immunohistochemistry

Immunohistochemical stains were performed on 5 *μ*m unstained sections from the tissue microarray blocks using the antibodies listed in Table 1. To retrieve the antigenicity, the tissue sections were treated at 100 °C in a steamer containing 10 mmol citrate buffer (pH 6.0) for 60 min. The sections were then immersed in methanol containing 0.3% hydrogen peroxidase for 20 min to block the endogenous peroxidase activity and were incubated in 2.5% blocking serum to reduce nonspecific binding. Sections were incubated for 90 min at 37 °C with primary antibodies at the dilutions specified in Table 1. Standard avidin–biotin immunohistochemical analysis of the sections was performed according to the manufacturer's recommendations (Vector Laboratories, Burlingame, CA, USA). Diaminobenzidine tetrahydrochloride was used as a chromogen, and haematoxylin was used for counterstaining.

Two observers independently analysed the immunostaining results and their intensities (JVP and HW). Positive and negative controls were used for each antibody on each slide. A third investigator (MJO) re-evaluated any cases with discrepancies between the two interpretations. Expression of PTEN, PMS2, hMLH-1, hMSH-2, hMSH-6, CK20, CK7, and CDX2 was considered positive if >10% of the tumour cells show immunoreactivity. The staining for HER2 was graded according to the guideline for HER2 testing in breast cancer with 2+ or 3+ membranous staining in 10% of the tumour cells as positive (Wolff *et al*, 2007). For VEGF-A and EGFR, both the percentage of positive tumour cells and the intensity of positive staining were graded. The percentage of staining was graded as follows: 0, staining in <1% of tumour cells; 1, staining in 1–20% of tumour cells; 2, staining in 20–50% of tumour cells; and 3, staining in 50–100% of tumour cells. The intensity of staining was graded as follows: 0, no staining; 1, weak staining; 2, moderate staining; and 3, strong staining. Composite grades for EGFR and VEGF-A was generated on a scale of 0–6 by adding grade for the percentage of cells stained to the grade for staining intensity (Goldstein and Armin, 2001).

### Microsatellite analysis

Representative slides from tumour and normal small bowel mucosa were obtained for each case and macro-dissection of the relevant tissue was performed. DNA was extracted by proteinase K digestion (Mirabelli-Primdahl *et al*, 1999). Genomic DNA was amplified by polymerase chain reaction (PCR) using fluorescently labeled and unlabeled primers for each of the five National Cancer Institute-recommended microsatellite markers (BAT-25, BAT-26, D2S123, D5S346, and D17S250). Polymerase chain reaction products were electrophoretically separated using an ABI 3130 analyser (Applied Biosystems, Foster City, CA, USA) and analysed using GeneMapper 4.0 software (Applied Biosystems). Samples with MSI at two or more loci were classified as MSI high, whereas samples with MSI at either one or none of the loci were classified as MSI low or microsatellite stable (MSS), respectively (Umar *et al*, 2004).

### Statistical analysis

Fisher's exact test was used to assess the association between various molecular markers. Overall survival curves were constructed using the Kaplan–Meier method, and the log-rank test was used to evaluate the statistical significance of differences and association with each molecular marker. The Cox proportional hazards regression model was used to assess the predictive effects of multiple covariates (including histology, stage, biomarkers, and demographical variables of the patients) on OS simultaneously. All analyses were performed using the S-PLUS software package (TIBCO Software Inc., Palo Alto, CA, USA).

## Results

### Clinicopathological characteristics of patients with SBA

The patient and tumour characteristics are summarised in Table 2. The ages of the patients ranged from 30 to 79 years, with a median of 54 years. The primary tumour site in the small bowel was the duodenum in 74% of cases.

### Immunophenotype of SBA

The most commonly expressed immunophenotypic marker was CDX2, observed in 38 patients (70%). The expression of CDX2 varied according to histological grade, with 100% of well-differentiated tumours and 58% of poorly differentiated tumours expressing CDX2. Expression of CK20 occurred in 31 (57%) patients, and expression of CK7 occurred in 17 (31%) patients. The most common combined cytokeratin expression pattern was CK20+/CK7− observed in 23 (43%) patients, followed by CK20−/CK7− observed in 15 (28%) patients (Table 3). There was no significant difference in the expression of CK7, CK20, and CDX2 between the duodenal and nonduodenal SBA or between the primary and metastatic tissue sites.

### Expression of DNA MMR enzymes and microsatellite instability

**Figure 1:**
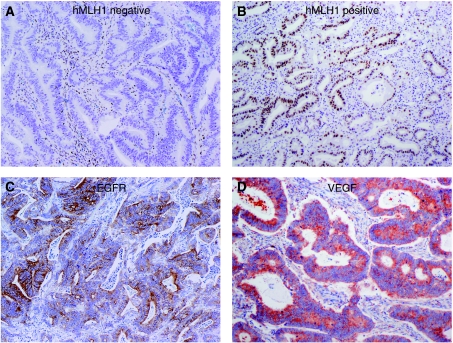
Representative immunohistochemical staining (original magnification × 100) of (**A**) absent MLH1 expression; (**B**) intact MLH1 expression; (**C**) epidermal growth factor receptor (EGFR) expression; and (**D**) vascular endothelial growth factor (VEGF) expression.

A total of 18 cases (35%) of SBA showed loss of expression of at least one MMR protein by immunohistochemical stains (Figure 1A and B). In all, three (6%) patients showed loss of MSH-2, six (11%) showed loss of MSH-6, 13 (24%) showed loss of PMS2, and 14 (26%) showed loss of hMLH-1. Loss of MMR protein expression generally occurred in two patterns, with combined loss of either MSH-2 with MSH-6 or PMS2 with hMLH-1. Because of the large number of patients who showed loss of MMR protein expression, we confirmed these results with MSI testing for these 18 cases. MSI testing was successful in 15 cases but could not be conducted in three cases because of lack of normal control tissue in two cases and lack of residual tumour tissue in one case. One case had an MSI-low phenotype and 14 cases had an MSI-high phenotype. Of the 15 cases showing MSI, 69% of the patients were younger than 55 years at the time of initial diagnosis. A comparison of patient and tumour characteristics between patients with intact MMR and patients with deficient MMR is presented in Table 4. Patients with deficient MMR tended to be younger and had a lower frequency of distant metastasis at presentation. As is observed in MMR-deficient adenocarcinomas of the colon, SBA with deficient MMR showed a significant lower frequency of CK20 expression than SBA with intact MMR (Table 4, *P*=0.02) (McGregor *et al*, 2004).

### Expression of VEGF-A, EGFR, HER2, and PTEN

Expression of VEGF-A, score 2–6, was observed in 50 (91%) patients and expression of EGFR, score 2–6, was observed in 36 (71%) patients (Figure 1C and D). The majority of cases showed high levels of expression with a score of 4–6 observed in 44 (81%) patients with VEGF-A and 26 (48%) patients with EGFR. Expression of HER2 was observed in only one patient, and no patients showed loss of PTEN expression.

### Survival analysis

**Figure 2:**
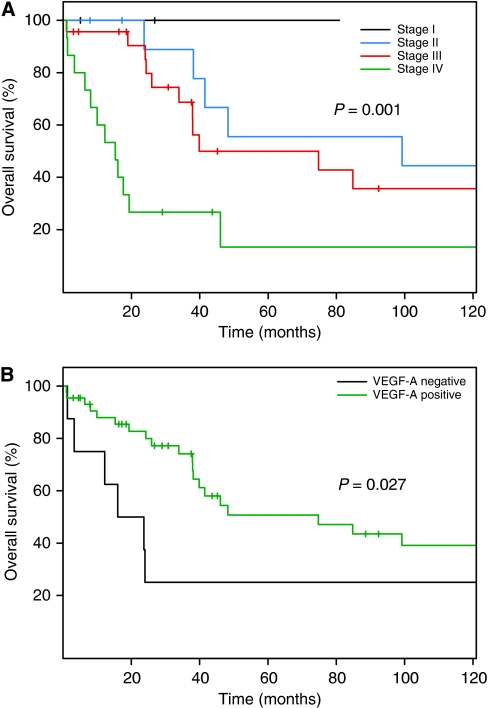
Kaplan–Meier overall survival plots according to (**A**) TNM classification and (**B**) expression of vascular endothelial growth factor A (VEGF-A), expression score 0–2 *vs* 3–6.

The median follow-up time was 45 months and the median survival was 46 months (95% CI 38–167 months). In the univariate analysis, TNM classification and VEGF-A expression correlated with OS (Figure 2A and B). Patients with low expression of VEGF-A (score of 0 or 1) had a shorter median OS, 19.9 months, compared with patients with high expression of VEGF-A, 74.8 months, *P*=0.027. The group of patients with low expression of VEGF-A also had a higher rate of metastatic disease (63% of patients) than patients with high VEGF-A expression (22% of patients), *P*=0.035. There was no difference in outcome by EGFR expression level or MMR protein expression status. On multivariate survival analysis, patients with higher TNM stage (hazard ratio (HR) 3.15, *P*=0.002) and poorly differentiated tumours (HR 2.95, *P*=0.049) showed significantly shorter OS.

## Discussion

Our study represents one of the largest clinicopathological studies of SBA and provides a clinically needed, robust immunophenotypic characterisation of this rare cancer as well as an exploration of the role of various oncogenic pathways in this tumour type. As in colorectal cancer, expression of CDX2, VEGF-A, and EGFR was observed in the majority of cases, but HER2/neu expression and loss of PTEN expression are rare oncogenic abnormalities in this cancer. Abnormalities in DNA MMR were common, occurring in over one-quarter of patients, but did not correlate with OS. In multivariate analysis, higher TNM stage and poorly differentiated histology were correlated with worse OS.

The expression pattern of cytokeratins, particularly CK20 and CK7, has proven clinical utility in determining the site of epithelial origin for various carcinomas. For colorectal cancer, a CK20+/CK7− expression pattern is observed in 75–94% of all patients (Loy *et al*, 1996; Rullier *et al*, 2000). In two previous large series of SBA, which included 23 (Lee *et al*, 2003) and 24 (Chen and Wang, 2004) patients, the reported rate of CK7 expression was 34 and 100%, respectively, and the rate of CK20 expression was 47 and 67%, respectively. Our study shows a low rate of CK7 expression in SBA patients, with the classic CK20+/CK7− colorectal cancer expression pattern observed in only 43% of patients. More clinically relevant was the high rate of CDX2 expression observed in this population (70%). This is similar to a previous report in which CDX2 positivity was observed in 4 out of 4 cases of adenocarcinoma of the duodenum and 18 of 30 cases of SBA (Werling *et al*, 2003; Zhang *et al*, 2007). The CDX2 is a highly sensitive marker for identifying adenocarcinomas of colonic origin, with expression observed in over 95% of colorectal cancers (Moskaluk *et al*, 2003; Werling *et al*, 2003). Colorectal cancer cases that are negative for CDX2 expression tend to be poorly differentiated tumours, and consistent with this finding, we observed that whereas 100% of well-differentiated tumours were CDX2 positive, only 58% of poorly differentiated tumours were CDX2 positive (Werling *et al*, 2003).

The reported incidence of abnormalities in MMR genes in SBA patients has been variable, ranging from 5 to 45% by microsatellite testing and from 0 to 26% by immunohistochemical staining of tumour samples (Hibi *et al*, 1995; Rashid and Hamilton, 1997; Blaker *et al*, 2002; Planck *et al*, 2003; Brueckl *et al*, 2004; Zhang *et al*, 2006). In our study, we saw a higher rate of defective MMR, with 35% of patients showing loss of MMR protein expression. This high rate was confirmed by MSI PCR analysis in all cases with available tissue. The high incidence of MSI in this study may relate with the young age of our study population. As in MSI colorectal cancer, SBA patients with MSI tended to be younger, have earlier-stage disease, and have a lower rate of CK20 expression (Gryfe *et al*, 2000; McGregor *et al*, 2004). In contrast with colorectal cancer, we did not see any evidence of an improved prognosis in the subset of patients with deficient expression of MMR proteins.

In an attempt to identify other oncogenic pathways involved in SBA, we characterised two members of the ErbB receptor tyrosine kinase family (EGFR and HER2), the pro-angiogenic protein VEGF-A, and the tumour suppressor protein PTEN. In one study of duodenal adenocarcinoma, HER2 was expressed in 9 of 16 cases and expression was correlated with a worse outcome (Zhu *et al*, 1996). In contrast, our study found HER2 expression in only 1 of 54 cases. The explanation for this discrepancy is unclear, although lack of specificity with the antibody used by Zhu *et al.* (1996) may be one explanation. The expression of EGFR was present in 71% of cases, which is similar to the rate of expression observed in colorectal cancer. As in colorectal cancer, the expression level of EGFR was not associated with changes in survival (McKay *et al*, 2002). Although limited numbers of patients have been tested, it seems that activating mutations in the *KRAS* oncogene are observed in approximately 40% of SBA patients, which is similar to the rate observed in colorectal cancer (Bamford *et al*, 2004). This finding, in conjunction with our finding of the high rate of EGFR expression in SBA, supports the clinical investigation of anti-EGFR therapy for SBA patients, with particular emphasis on patients without activating mutations in the *KRAS* oncogene.

We saw expression of VEGF in 91% of patients with SBA. This correlates with another study that found universal expression of VEGF-A RNA in a small number of SBA patients (von Rahden *et al*, 2006). Interestingly, our data showed worse survival in those patients with low levels of VEGF-A expression. This subgroup of patients had a much higher rate of metastatic disease, which may suggest the presence of alternative pro-invasion and pro-metastatic signalling pathways. Fibroblast growth factor, platelet-derived growth factor, and notch-mediated signalling are all examples of pro-angiogenic stimuli that are not dependant on VEGF ligand expression (Ellis and Hicklin, 2008). The importance of alternative angiogenic pathways has recently been shown by work in mouse model systems, in which greater invasiveness and higher rates of distant metastases were observed after the genetic ablation of the *VEGF-A* gene (Paez-Ribes *et al*, 2009). Given the limited sample size of this study, further investigation to verify the effect of the VEGF pathway on the outcome in small bowel adenocarcinoma is needed.

The phosphatase and tensin homologue mutated on chromosome 10 is a potent tumour suppressor that is commonly lost in a number of sporadic tumours and inherited loss of PTEN results in Cowden's syndrome. This syndrome is characterised by multiple hamartomas that occur throughout the gastrointestinal tract, including the small bowel. In mouse models, the loss of PTEN in the context of APC deficiency accelerates tumourigenesis through increased activation of AKT, leading to the rapid development of adenocarcinoma (Dahia, 2000). In colorectal cancer, inactivation of PTEN is primarily observed in patients with MSI tumours, occurring in 14–19% of cases (Zhou *et al*, 2002; Goel *et al*, 2004). We did not identify any patients with loss of PTEN expression, which may suggest a less critical role for the PI3K/AKT pathway in carcinogenesis in the small bowel. Similarly, as the majority of PTEN inactivation in colorectal cancer results from *pten* gene methylation, this result may suggest a more limited role of hypermethylation in SBA compared with colorectal cancer.

In summary, this study characterised the immunophenotype, DNA MMR gene status and the expression of a number of candidate oncogenic proteins in a large group of SBA. The high levels of VEGF-A and EGFR expression in SBA provide a rationale for the exploration of therapies targeting these oncogenic pathways in this rare tumour type.

## Figures and Tables

**Table 1:** Antibodies used

**Antibody**	**Clone**	**Titre**	**Company**
Anti-CDX2	CDX-88	1 : 50	Biogenex, San Ramon, CA, USA
Anti-CK7	OVT-TL 12/30	1 : 100	Dako, Carpinteria, CA, USA
Anti-CK20	KS20.8	1 : 4000	Dako, Carpinteria, CA, USA
Anti-EGFR	31G7	1 : 50	Invitrogen, Inc., Carlsbad, CA, USA
Anti-HER2	neu Ab8	1 : 300	Labvision, Inc., Fremont, CA, USA
Anti-MLH-1	G168-15	1 : 25	BD Biosciences, San Jose, CA, USA
Anti-MSH-2	FE11	1 : 100	Calbiochem, Inc., Gibbstown, NJ, USA
Anti-MSH-6	44	1 : 300	BD Biosciences, San Jose, CA, USA
Anti-PMS2	A16-4	1 : 125	BD Biosciences, San Jose, CA, USA
Anti-PTEN	28H6	1 : 100	Novacastra, Bannockburn, IL, USA
Anti-VEGF-A	VEGF A20	1 : 10	Santa Cruz Biotechnology, Santa Cruz, CA, USA

Abbreviations: CDX2=caudal type homeobox transcription factor 2; EGFR=epidermal growth factor receptor; MLH-1=MutL homologue 1; MSH-2=MutS homologue 2; PMS2=post meiotic segregation increased 2; PTEN=phosphatase and tensin homologue mutated on chromosome 10; VEGF-A=vascular endothelial growth factor A.

**Table 2:** Patient and tumour characteristics

**Characteristic**	**No. (%) of patients**
*Age*	
Median	54 years
Range	30–79 years
	
*Sex*	
Men	34 (63)
Women	20 (37)
	
*Ethnicity*	
Caucasian	43 (80)
Black	5 (9)
Other	6 (11)
	
*Initial stage*	
I	5 (9)
II	11 (20)
III	23 (43)
IV	15 (28)
	
*Primary tumour site*	
Duodenum	40 (74)
Jejunum	4 (7)
Ileum	8 (15)
Small bowel NOS	2 (4)
	
Mucinous histology	15 (28)
	
*Histological grade*	
Well differentiated	4 (8)
Moderately differentiated	38 (70)
Poorly differentiated	12 (22)

**Table 3:** Number of patients with each immunophenotype

	**CDX2**	**CK20**	**CK7**	**CK20+/CK7−**	**CK20+/CK7+**	**CK20−/CK7−**	**CK20−/CK7+**
Positive, %	70	57	31	43	15	28	13
Negative, %	30	43	69	57	85	72	87

**Table 4:** Patient and tumour characteristics stratified by intact or deficient DNA mismatch repair (MMR) status

	**Number of patients (%)**		
	**Intact MMR**	**Deficient MMR**	***P*-value**
Patient number	36	18	
			
*Age*			0.63
Median	56	49	
Range	30–78	37–79	
			
*Sex*			0.99
Men	23 (64)	11 (61)	
Women	13 (36)	7 (39)	
			
*Stage*			0.5
I	4 (11)	1 (6)	
II	7 (19)	4 (22)	
III	13 (36)	10 (56)	
IV	12 (33)	3 (17)	
			
*Primary tumour site*			0.3
Duodenum	27 (75)	13 (72)	
Jejunum	4 (11)	4 (22)	
Ileum	4 (11)	0 (0)	
Small bowel NOS	1 (3)	1 (6)	
Mucinous histology	9 (25)	6 (33)	0.54
			
*Histological grade*			0.18
Well differentiated	2 (6)	2 (11)	
Moderately differentiated	19 (53)	13 (72)	
Poorly differentiated	9 (25)	3 (17)	
			
CK 20 expression	25 (69)	6 (33)	0.02
CK 7 expression	11 (31)	6 (33)	0.99
CDX-2 expression	26 (72)	12 (67)	0.76
